# Peak urea level, leukocyte count and use of invasive ventilation as risk factors of mortality in acute pancreatitis: A retrospective study

**DOI:** 10.1371/journal.pone.0216562

**Published:** 2019-05-10

**Authors:** Chao-Nan Liu, Si Chen, Hao Chen, Li Yue, Li-Qin Ling, Chang-Wei Chen, Lei Du, Jing Zhou

**Affiliations:** 1 Department of Laboratory Medicine, West China Hospital, Sichuan University, Chengdu, China; 2 Workplace Safety & Insurance Board, Toronto, Ontario, Canada; 3 Department of Anesthesiology and Translational Neuroscience Center, West China Hospital, Sichuan University, Chengdu, China; University of Szeged, HUNGARY

## Abstract

**Background:**

Acute pancreatitis (AP) is associated with high complications. Early, reliable prediction of mortality may improve patient management.

**Methods:**

We retrospectively reviewed medical records of 1,599 patients with AP treated at a single large hospital in southwest China. Models to predict mortality were derived from a subset of 1,062 patients (development dataset), and the models were then validated in the remaining 537 patients (validation dataset). Independent risk factors and prediction models for mortality were identified using logistic regression.

**Results:**

A total of 33 patients in the development dataset and 13 in the validation dataset died during hospitalization. Independent risk factors for mortality were found to be plasma urea levels, glucose levels and platelet counts at admission; as well as peak urea levels, leukocyte counts and use of invasive ventilation during hospitalization. Based on the development dataset, a mortality prediction model based only on urea level at admission gave an area under the curve (AUC) of 0.81, which did not significantly improve by incorporating glucose level or platelet count at admission. Significantly better was a model taking into account three in-hospital parameters: peak urea level, leukocyte count and use of invasive ventilation (AUC 0.97).

**Conclusions:**

While mortality of AP patients can be predicted reasonably well based only on urea values at admission, predictions are more reliable when they take into account in-hospital data on peak urea level, leukocyte count and use of invasive ventilation.

## Introduction

Acute pancreatitis (AP) involves sudden abdominal inflammation, which leads to millions of hospitalizations annually around the world [1,2]. Approximately 15–20% of patients die from this disease in the intensive care unit [3,4]. Early, reliable prediction of which patients are at higher risk of mortality may help improve treatment and management of AP. The Acute Physiology and Chronic Health Evaluation (APACHE) [5] instrument has been used to predict AP-related mortality based on weighted scoring of three sets of variables measured within a few hours of hospital admission. Slightly simpler scoring systems have been developed [6–8], but they have not been widely adopted, perhaps reflecting their complexity and relative unreliability.

One of the simplest models to predict AP-related mortality [9, 10] takes into account only plasma urea level at admission, meaning that it can be applied to patients even when the time from AP onset to admission is unknown. This model showed an ability to predict mortality in Caucasian AP patients with an area under the receiver operating characteristic curve (AUC) of 0.79, comparable to the AUC of 0.83 obtained with the much more complex APACHE II instrument. The power of this simple model was improved by incorporating the increase in urea level during the first 24 h after admission (AUC 0.89) as well as during the first 48 h after admission (AUC 0.90). These results suggest that reliable prediction of AP-related mortality may require taking into account risk factors of AP-related mortality after admission [11].

Here we wanted to examine whether urea levels at admission can predict AP-related mortality in a Chinese population of AP patients, and whether the predictive power can be improved by incorporating in-hospital patient data into the model. Therefore we retrospectively analyzed data for AP patients treated at our large hospital in southwest China over a five-year period.

## Materials and methods

### Patients

This retrospective study included a consecutive series of patients admitted for AP at West China Hospital of Sichuan University between January 1, 2011 and December 31, 2015. In accordance with the revised Atlanta Classification (2012),[12] patients were diagnosed with AP if they presented with two or more of the following: (1) abdominal pain consistent with acute pancreatitis (acute onset of a persistent, severe, epigastric pain often radiating to the back); (2) serum levels of amylase and/or lipase ≥3 times the upper limit of normal; and (3) characteristic features on contrast-enhanced computed tomography (CECT) and, less commonly, magnetic resonance imaging (MRI) or transabdominal ultrasonography. Patients were excluded if they were younger than 18 years, or had been admitted to our hospital more than 48 h after AP onset. All data were fully anonymized before accessed. This study was approved by the Ethics Committee of West China Hospital of Sichuan University.

### Data collection

Working with the hospital central database, two authors independently extracted data on patient characteristics and clinical variables, including laboratory data, treatments and outcomes. The two data sets were checked against each other, and discrepancies were resolved through discussion and closer examination of the original data. Serum amylase and other biochemical tests as well as blood counts were performed within 2 h of admission, as per standard procedure at our hospital. Blood counts were performed every 1–2 days thereafter. Other biochemical indicators were assayed at the discretion of the attending physician. Index-time curves were plotted to determine when these biochemical indices reached a maximum or minimum. Data were also collected on patients who showed altered mental status [13] during AP progression.

Data were collected on hospital procedures that might have influenced the prognosis of AP. These included invasive ventilation, defined as involving endotracheal intubation or tracheotomy; noninvasive ventilation; surgery; and dialysis. Invasive ventilation, which is much easier to evaluate than respiratory function, served as an indicator of damaged pulmonary gas exchange and/or respiratory muscle function.

### Statistical analysis

The full dataset was divided into two subsets. The development dataset, used to construct risk models of mortality, comprised patients treated from January 1, 2011 to December 31, 2012 and from January 1, 2014 to March 31, 2015. The resulting risk models were validated using the remaining data (validation dataset) covering one-third of the study period, corresponding to patients treated from January 1 to December 31, 2013 and from April 1, 2015 to December 31, 2015.

Data in the development dataset were reported as mean ± standard deviation and analyzed by unpaired or revised Student’s t test if they showed a normal distribution based on Shapiro-Wilk tests, or as median (interquartile range) and analyzed using the rank sum test if they showed a non-normal distribution. Data for categorical variables were expressed as percentages, and inter-group differences were analyzed using chi-squared or Fisher’s exact tests.

All variables that differed significantly between groups were considered as potential risk or confounding factors affecting mortality, and so were included in forward univariate logistic regression to identify mortality risk factors at admission and during hospitalization. Variables significant at the p < 0.05 level in univariate regression were then entered in multivariate regression. Analysis was adjusted by other indices when one index was used to estimate risk. The resulting odds ratio (OR) represents the predicted change in risk per unit increase in the predictor.

Mathematical models to predict AP-associated mortality were developed as follows. All variables significant at the p < 0.05 level in multivariate logistic regression were entered into binary logistic regression, and non-significant variables were eliminated from the model one at a time. Each time that a variable was added, the stability of the model was checked by examining the p value and β value [β = ln (OR)]. We constructed a multimarker score H = (β1×biomarker A) + (β2×biomarker B) + (β3×biomarker C), where the coefficients β1, β2 and β3 were estimated in the binary logistic regression. The correspondence between model predictions and observed data was assessed using the Hosmer-Lemeshow chi-squared test.

Formulas based on data at admission were generated by taking into account demographic characteristics, disease history and indices calculated within 2 h of admission. To test whether incorporating post-admission data might improve the predictive power of the formulas, we also generated formulas based on the maximum or minimum values of laboratory tests, altered mental status and special procedures.

To assess the predictive power of scores derived from formulas or from urea level, receiver operating characteristic (ROC) curves were generated against the development dataset. Patients classified as low- or high-risk were defined based on the optimal ROC threshold value, which was determined from Youden’s index based on specificity and sensitivity. High-risk patients were those with scores above the threshold value; others were low-risk. Then an adjusted OR and 95% confidence interval (95%CI) referring to high-risk patients relative to low-risk ones was obtained by logistic regression. Positive and negative predictive values (PPV, NPV) were assessed for scores and urea levels using the validation dataset. PPV assesses how well the model predicts patients who actually experience the complication. The numerator gives the number of those high-risk patients who actually experienced the mortality, while the denominator gives the number of patients classified by the model as being at high risk (scores > threshold value). NPV assesses how well the model predicts patients who don’t experience the complication. The numerator gives the number of those low-risk patients who actually don’t experienced the mortality, while the denominator gives the number of patients classified by the model as being at low risk (scores < threshold value).

All statistical analyses were performed using SPSS 19.0 (IBM, Chicago, IL, USA) and MedCalc 15.2.2 (Mariakerke, Belgium). In all analyses, p < 0.05 was considered significant.

## Results

**Fig 1:**
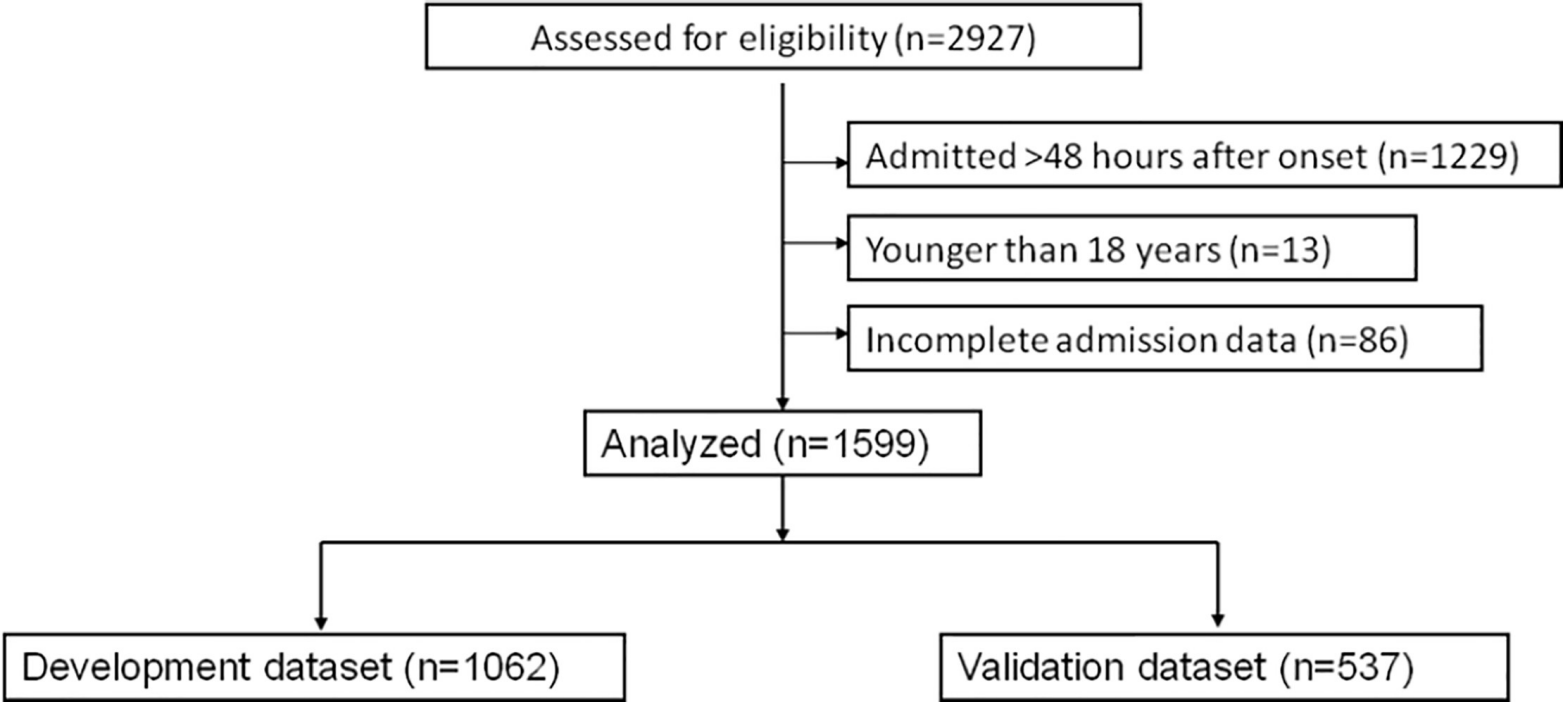
Flow diagram of patients.

Of the 2,927 potentially eligible patients treated at our hospital, 1,328 were excluded, and the remaining 1,599 patients were included in the analysis (Fig 1). Of these, 1,062 were assigned to the development dataset to construct risk models, while the remaining 537 were assigned to a validation dataset to assess the quality of the models. Baseline characteristics of both groups of patients are shown in Table 1. The cause of AP was not possible to determine definitively in most of our sample because of the short disease course: the disease was related to food or was not linked to any obvious cause in 977 patients (92%), it was linked to cholangitis in 21 patients (2%), and it was related to alcohol in the remaining 64 patients (6%).

**Table 1:** Clinicopathological characteristics of acute pancreatitis patients in the development dataset^a^^b^.

Variable	Died (n = 33)	Survived (n = 1029)	p
**Demographic features**			
Age, yr	53.3±15.6	47.1±13.9	0.012
Male, n (%)	17 (51.5)	614 (59.7)	0.348
Smoking, n (%)	7 (21.2)	408 (39.7)	0.033
Alcohol, n (%)	5 (15.2)	223 (21.7)	0.369
Weight, kg	68.3±10.4	68.9±12.5	0.843
Height, cm	163±7	164±8	0.373
BMI, kg/m^2^	26.1±2.0	25.4±3.6	0.493
Diabetes, n (%)	6 (18.2)	191 (18.6)	0.956
Stroke history, n (%)	1 (3.0)	5 (0.5)	0.173
Hypertension, n (%)	10 (30.3)	179 (17.4)	0.056
Hyperlipidemia, n (%)	10 (30.3)	415 (40.3)	0.247
Coronary heart disease, n (%)	2 (6.1)	32 (3.1)	0.656
ACEI/ARB, n (%)	1 (3.0)	43 (4.2)	1.000
β-blocker, n (%)	1 (3.0)	13 (1.3)	0.359
Calcium antagonists, n (%)	6(18.2)	79 (7.7)	0.62
Diuretics, n (%)	2 (6.1)	12 (1.2)	0.067
Insulin, n (%)	4 (12.1)	116 (11.3)	1.000
Anti-platelet agents, n (%)	0 (0.00)	6 (0.6)	1.000
Statins, n (%)	0 (0.00)	36 (3.5)	0.545
**At admission (within 48 h of symptom onset)**			
Alanine transaminase, U/L	34 (22,82)	37 (21,82)	0.710
Glucose, mmol/L	13.17±6.01	9.66±4.99	<0.05
Total bilirubin, μmol/L	19.9 (11.6,33.7)	17.5 (11.5,27.1)	0.423
Conjugated bilirubin, μmol/L	7.80 (4.7,22.8)	6.4 (4.0,11.8)	0.080
Lipase, 1×10^3^ U/L	1.24 (0.85,2.54)	0.68 (0.25,1.32)	<0.05
Amylase, 1×10^3^ U/L	1.44 (0.71,2.12)	0.46 (0.17,1.08)	<0.05
Urea, mmol/L	10.00±5.01	5.35±2.79	<0.05
Aspartate transaminase, U/L	71 (39,166)	37 (23,86)	<0.05
Creatinine, μmol/L	180±143	79±50	<0.05
Platelets, ×10^9^/L	131±63	159±69	0.020
Leukocytes, ×10^9^/L	15.4±7.4	13.6±5.5	0.175
**In hospital**^**c**^			
Alanine transaminase peak, U/L	135 (40,344)	51 (28,115)	<0.05
Glucose peak, mmol/L	17.12±6.46	11.07±5.95	<0.05
Total bilirubin peak, μmol/L	60.4 (22.0,124.9)	22.8 (16.4,35.6)	<0.05
Conjugated bilirubin peak, μmol/L	48.3 (11.3,107.8)	9.4 (5.9,18.5)	<0.05
Lipase peak, U/L	1.49 (0.93,2.54)	0.74 (0.30,1.37)	<0.05
Amylase peak, U/L	1.77 (0.97,2.31)	0.51 (0.20,1.18)	<0.05
Urea peak, mmol/L	22.17±13.38	6.67±3.50	<0.05
Aspartate transaminase peak, U/L	263 (70,1234)	50 (29,109)	<0.05
Creatinine peak, μmol/L	326±318	89±57	<0.05
Leukocyte peak, ×10^9^/L	26.7±13.0	15.7±6.6	<0.05
Platelet count nadir, ×10^9^/L	81±71	134±56	<0.05
Altered mental status, n (%)^d^	15 (45.5)	25 (2.4)	<0.05
**Interventions, n (%)**			
Invasive ventilation	25 (75.8)	41 (4.0)	<0.05
Noninvasive ventilation	22 (66.7)	144 (14.0)	<0.05
Surgery	15 (45.5)	135 (13.1)	<0.05
Dialysis	14 (42.4)	14 (1.4)	<0.05
**Hospital stay, d**	28±44	13±11	0.063

^a^ Continuous data were reported as mean ± SD (normal distribution), or median (interquartile range) (skewed distribution). Inter-group differences were assessed for significance using the t test (normal distribution) or nonparametric rank sum test (skewed distribution). Data for categorical variables were reported as incidence (%), and inter-group differences were assessed using the chi-squared or Fisher’s exact tests.

^b^ A total of 1,062 patients were included in the development dataset (see Methods).

^c^ Index-time curves were plotted for biochemical indices to identify peak and nadir values.

^d^ Altered mental status included delirium, somnolence, lethargy and coma.

**Abbreviations**: ACEI, angiotensin-converting enzyme inhibitor; ARB, angiotensin receptor blocker; BMI, body mass index.

### Model building

#### Independent risk factors at admission

**Fig 2:**
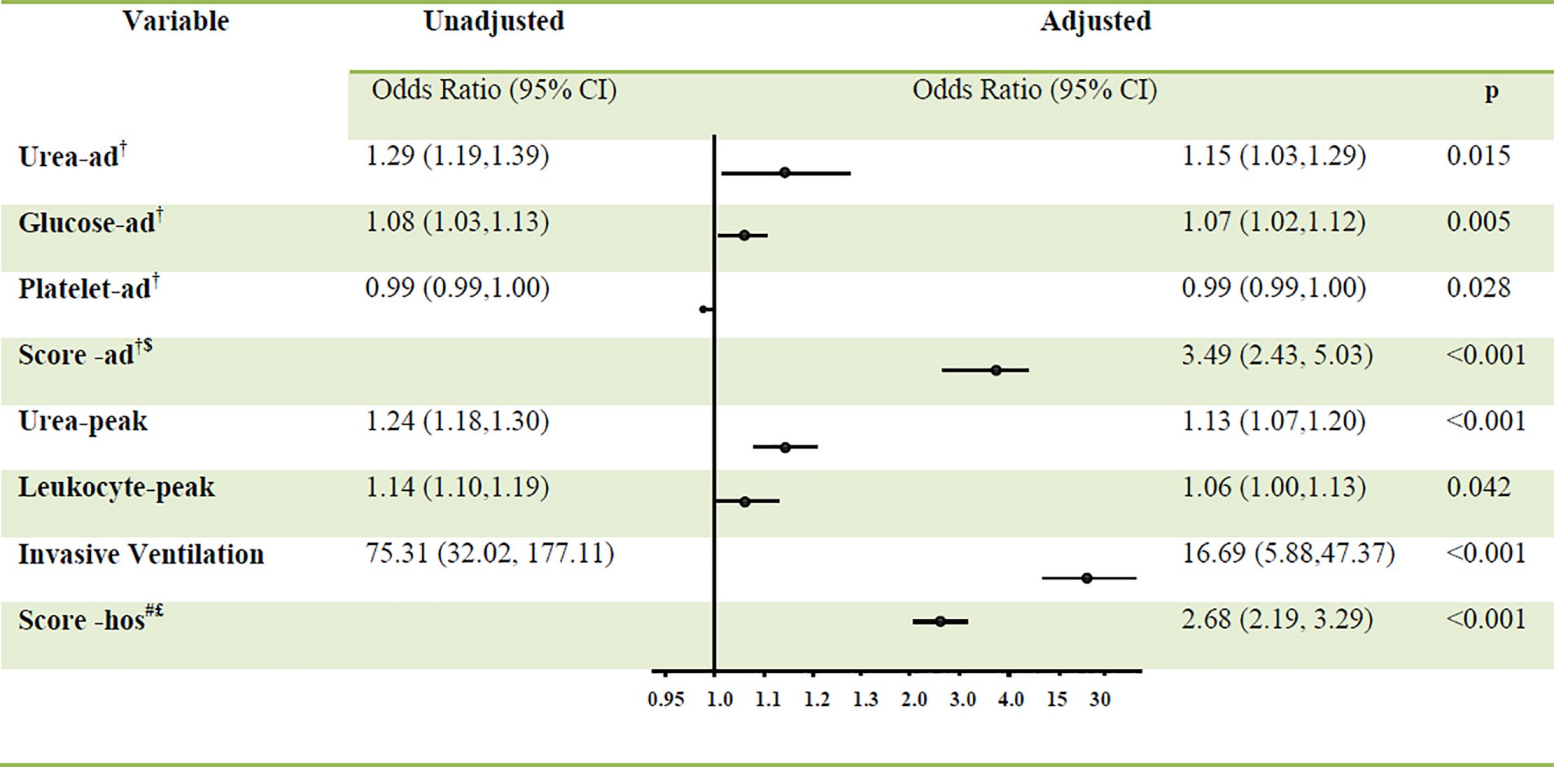
Univariate and multivariate logistic regression to identify risk factors of mortality due to acute pancreatitis. †Ad, at admission. $ Score from the formula Y = (0.069×Glu-ad) +(0.141×Urea-ad)–(0.008×PLT-ad) # Hos, during hospitalization. £ Score from the formula Y = (0.121×Urea-peak) + (0.060×WBC-Peak) + (2.815×IMV). Abbreviations: 95%CI, 95% confidence interval; Peak, maximal value of a laboratory test.

A total of 33 patients (3.11%) in the development dataset and 13 (2.23%) in the validation dataset died during hospitalization. The validation and development datasets showed similar distributions for variables of interest. In the development dataset, patients who died were older (53 vs 47 years, p = 0.012) and less likely to smoke cigarettes (21.2 vs 39.7%, p = 0.033) than those who survived. Patients who died also had higher levels at admission of blood glucose, lipase, amylase, urea, aspartate transaminase and creatinine, as well as lower platelet counts. In multivariate regression, blood glucose, urea level and platelet count at admission were independent risk factors for death, and they were retained in forward-selection models to create risk models of mortality (Fig 2).

#### Independent risk factors during hospitalization

In the development dataset, patients who died had higher peak levels of alanine transaminase, glucose, total bilirubin, conjugated bilirubin, lipase, amylase, urea, aspartate aminotransferase, creatinine, and leukocyte counts than patients who survived. Patients who died also had lower nadir platelet counts and higher incidence of altered mental status and special procedures (Table 1). In multivariate logistic regression, peak urea levels, leukocyte count, and use of invasive ventilation were independent risk factors of death. These factors were incorporated into the formula to calculate hospital scores (Fig 2).

### Model validation

Models developed based on the independent risk factors identified above were assessed for predictive power using the data in the validation dataset. Patients with risk scores above the threshold values were more likely to die: PPV was 8.4% and NPV was 99.3% based on scores at admission, compared to 24.4% and 99.6% based on scores during hospitalization.

#### Models based only on risk factors at admission

Urea levels at admission were 4.83 mmol/L (95%CI, 1.61 to 11.62) among patients who survived and 9.82 mmol/L (95%CI, 2.88 to 22.40; p < 0.001) among non-survivors; the corresponding scores at admission (score-ad) were 0.09 (95%CI, -1.38 to 1.70) and 1.28 (95%CI, -0.40 to 2.71; p < 0.001). Risk of mortality increased in a stepwise manner with increasing urea levels at admission (OR 1.15, 95%CI 1.03 to 1.29, p = 0.015) and score-ad (OR 3.49, 95%CI 2.43 to 5.03, p < 0.001).

**Fig 3:**
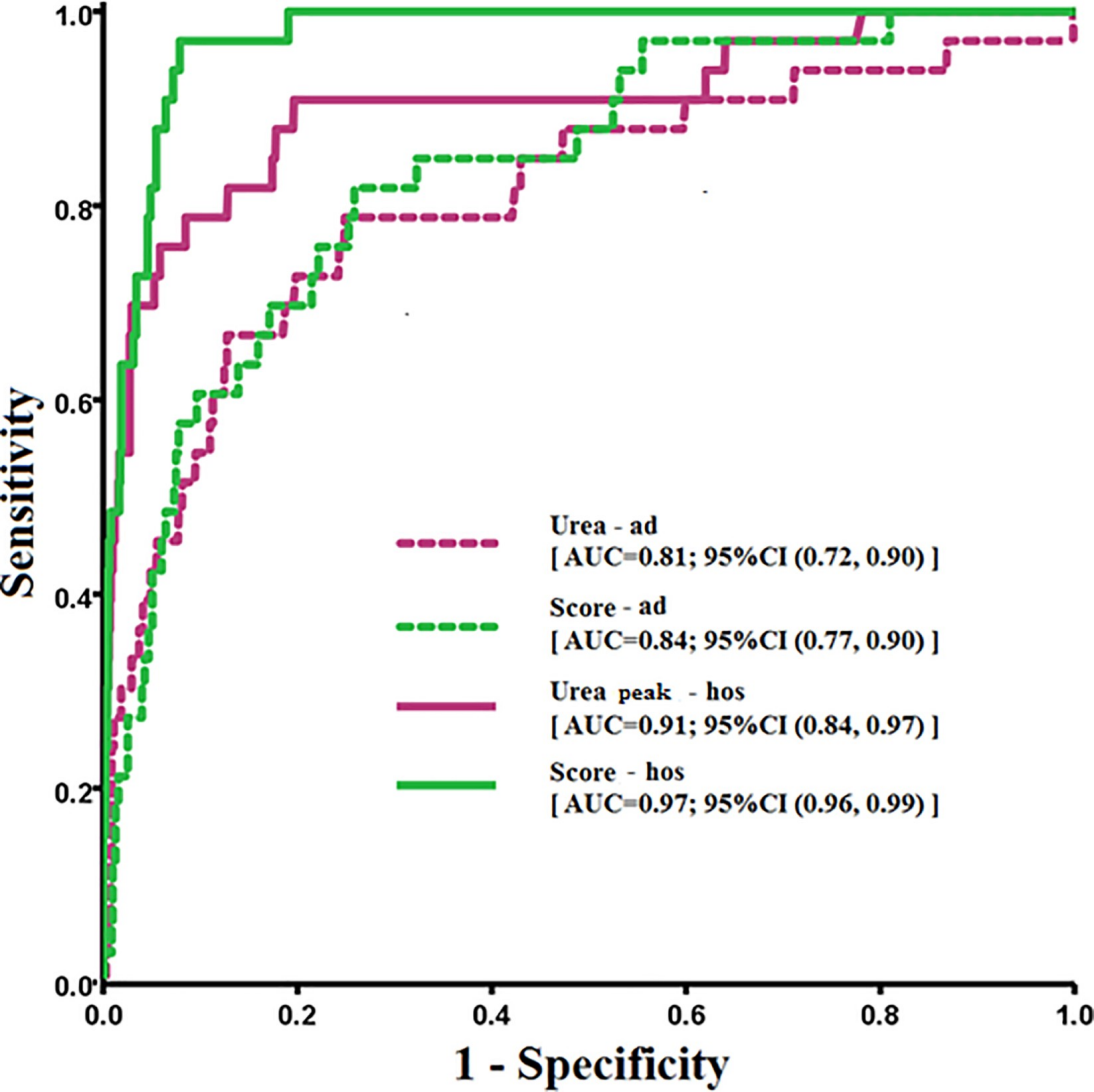
Receiver operating characteristic curve for urea level or scores based on data at admission or during hospitalization. Curves were calculated based on the formulas in Table 2. Predictions were based on data at admission (Ad, dotted lines) or on data during hospitalization (Hos, solid lines). Data based on urea levels are shown in purple; data based on scores, in green.

AUC for the model based solely on urea level at admission was 0.81 (95%CI 0.72 to 0.90). AUC did not improve significantly (p = 0.472) after incorporating glucose levels and platelet counts at admission (0.84, 95%CI 0.77 to 0.90; Fig 3). Based on Youden’s index, threshold values from ROC curves were 6.43 mmol/L for urea level at admission and 0.567 for score-ad. Sensitivity and specificity were 82.6% and 75.7% for urea level, similar to 80.4% and 75.9% for score-ad (Table 2).

**Table 2:** Accuracy of formulas to predict AP-related mortality^a^.

	Sensitivity	Specificity
Urea-ad	82.6%	75.7%
Score-ad	80.4%	75.9%
Urea-peak	90.3%	79.8%
Score-hos	93.5%	92.9%

^a^ Based on threshold values obtained using Youden’s index: Urea-ad, 6.43; Score-ad, 0.567; Urea-hos, 8.02; Score-hos, 2.916. Ad, at admission; Hos, during hospitalization.

#### Models based on risk factors at admission and during hospitalization

In the development dataset, peak urea levels during hospitalization were 6.00 mmol/L (95%CI 2.91 to 15.68) among patients who survived and 16.48 mmol/L (95%CI 5.34 to 50.96) among those who died. The corresponding scores during hospitalization (score-hos) were 1.62 (95%CI 0.87 to 5.21) and 5.80 (95%CI 2.19 to 11.68). AUC for a model predicting mortality based only on peak urea during hospitalization was 0.91 (95%CI 0.84 to 0.97), significantly higher than the AUC for a model based only on urea level at admission (p < 0.001). AUC for a score-hos including peak urea levels, leukocyte counts and use of invasive ventilation was 0.97 (95%CI 0.96 to 0.99), significantly higher than that based only on score-ad (p = 0.025) or only on peak urea level during hospitalization (p = 0.038; Fig 3).

Defining low and high mortality risk based on a threshold peak urea level during hospitalization of 8.02 mmol/L led to high sensitivity of 90.3% but medium specificity of 79.8%. The threshold score-hos was 2.916, which offered sensitivity of 93.5% and specificity of 92.9%. These values were higher than those obtained based only on peak urea level (Table 2).

## Discussion

Various scoring systems for predicting risk of AP-related mortality have been developed, and they show similar predictive power (AUC 0.79–0.84) in Caucasian populations [10]. In the present study, we found that as in Caucasian AP patients, urea levels within 48 h after AP onset can predict mortality in Chinese AP patients reasonably well (AUC 0.81). This predictive power was not substantially improved by expanding the model to include glucose levels and platelet counts at admission. In contrast, predictive power was significantly improved by adding data on three in-hospital parameters: peak urea level, leukocyte count, and use of invasive ventilation. Our results suggest that the most reliable prediction of AP-related mortality requires taking into account these three in-hospital indicators that capture AP progression. The simplified model that we present here may improve the management of AP patients from admission to discharge. It can be calculated in a straightforward way from routinely available data so that it can easily be used even in primary care settings.

The AUC of 0.81 achieved here with Chinese patients using only urea level at admission is similar to the 0.79 reported in Caucasian patients. [9,10] In fact, our predictive power is similar to that reported for several more complex models: APACHE II, AUC 0.83; [5] BISAP, AUC 0.82; [6] BALI, AUC 0.82;[7] and POP, AUC 0.84 [8]. We were unable to improve the predictive power of urea level by adding glucose level and platelet count at admission (AUC 0.84; 95%CI 0.77 to 0.90). These results suggest that in the absence of a better alternative, plasma urea level at admission serves as a reasonably good early predictor of AP-related mortality.

We were able to substantially improve on the predictive power based on urea level at admission simply by incorporating peak urea level during hospitalization (AUC 0.91, 95%CI 0.84 to 0.97, p = 0.025 vs urea level at admission). Adding this parameter improved sensitivity from 82.6% to 90.3%, although specificity improved only slightly from 75.7% to 79.8%. This improvement in predictive power through inclusion of in-hospital data echoes results from Wu et al [9], who achieved an AUC of 0.84 (95%CI 0.79 to 0.90) by incorporating data on changes in urea levels during the first 24 h after admission.

We found that including even more in-hospital data substantially improved predictive power. A model taking into account the three in-hospital parameters of peak urea level, leukocyte count and use of invasive ventilation gave an AUC of 0.97, sensitivity of 93.5% and specificity of 92.9%. These results suggest that including commonly measured data on a small number of patient characteristics during hospitalization can reliably predict AP-related mortality.

This study involved a relatively small number of deaths in a patient population from a single medical center, albeit one of the largest in China that draws patients from a large geographic area. These factors increase the risk of bias in the study. Our model focused on factors routinely analyzed in our hospital, which means that we neglected some factors that may influence AP-related mortality and so may improve our minimal model here; these factors include levels of some inflammatory factors, such as C-reactive protein and interleukin-6 [7], and levels of arterial blood gases [8]. Our model may also be improved by taking into account aspects of AP morbidity.

In conclusion, our results suggest that urea level at admission is a reasonably good predictor of mortality in AP patients, but that much better prediction is obtained by incorporating in-hospital data on peak urea levels, leukocyte count and use of invasive ventilation. We suggest this simple scoring system to improve management of AP patients. It may also be worth investigating whether this straightforward scoring system can predict mortality in patients with diseases other than AP.

## Supporting information

S1 Table The partial data of validation dataset. (XLS)
